# Tryptophan Challenge in Healthy Controls and People with Schizophrenia: Acute Effects on Plasma Levels of Kynurenine, Kynurenic Acid and 5-Hydroxyindoleacetic Acid

**DOI:** 10.3390/ph15081003

**Published:** 2022-08-15

**Authors:** Korrapati V. Sathyasaikumar, Francesca M. Notarangelo, Deanna L. Kelly, Laura M. Rowland, Stephanie M. Hare, Shuo Chen, Chen Mo, Robert W. Buchanan, Robert Schwarcz

**Affiliations:** Maryland Psychiatric Research Center, Department of Psychiatry, University of Maryland School of Medicine, Baltimore, MD 21228, USA

**Keywords:** cognition, cytokines, drug development, kynurenine pathway, serotonin

## Abstract

The pivotal tryptophan (TRP) metabolite kynurenine is converted to several neuroactive compounds, including kynurenic acid (KYNA), which is elevated in the brain and cerebrospinal fluid of people with schizophrenia (SZ) and may contribute to cognitive abnormalities in patients. A small proportion of TRP is metabolized to serotonin and further to 5-hydroxyindoleacetic acid (5-HIAA). Notably, KP metabolism is readily affected by immune stimulation. Here, we assessed the acute effects of an oral TRP challenge (6 g) on peripheral concentrations of kynurenine, KYNA and 5-HIAA, as well as the cytokines interferon-γ, TNF-α and interleukin-6, in 22 participants with SZ and 16 healthy controls (HCs) using a double-blind, placebo-controlled, crossover design. TRP raised the levels of kynurenine, KYNA and 5-HIAA in a time-dependent manner, causing >20-fold, >130-fold and 1.5-fold increases in kynurenine, KYNA and 5-HIAA concentrations, respectively, after 240 min. According to multivariate analyses, neither baseline levels nor the stimulating effects of TRP differed between participants with SZ and HC. Basal cytokine levels did not vary between groups, and remained unaffected by TRP. Although unlikely to be useful diagnostically, measurements of circulating metabolites following an acute TRP challenge may be informative for assessing the in vivo efficacy of drugs that modulate the neosynthesis of KYNA and other products of TRP degradation.

## 1. Introduction

**Figure 1:**
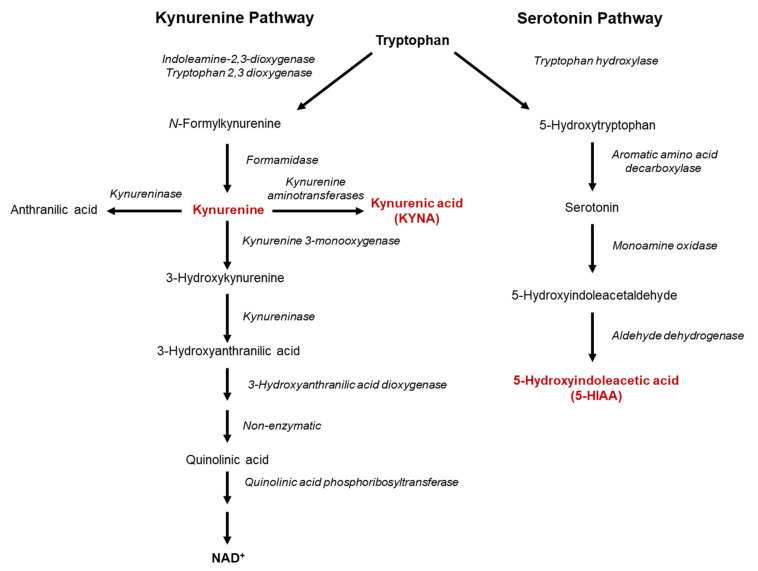
Tryptophan degradation through the kynurenine and serotonin pathways.

In mammals, the essential amino acid L-tryptophan (TRP) is not only used for protein synthesis but is also converted to a number of metabolites with noteworthy biological activities in the brain and elsewhere [1,2,3] (Figure 1). Remarkably, the major neurotransmitter serotonin, which regulates sleep, mood and appetite and has been plausibly linked to several brain disorders, including bipolar disorder, depression, insomnia, pain and aggression [4,5,6], is a quantitatively minor metabolic product of TRP in the periphery [7,8,9]. The vast majority of ingested TRP is converted via the kynurenine pathway (KP) [10], eventually leading to the production of the ubiquitous cofactor nicotinamide adenine dinucleotide (NAD^+^). The KP is responsible for the formation of several neuroactive compounds, which are increasingly understood to play significant roles in the physiology and pathology of the central nervous system [11,12,13]. Among these, the neuroinhibitory compound kynurenic acid (KYNA), which is formed from kynurenine, mainly by irreversible enzymatic transamination, as well as the neuroexcitatory metabolite quinolinic acid, has been studied most extensively, although several other pathway products may also function as endogenous neuromodulators (cf. [14] for a recent review).

KYNA, which notably does not normally cross the blood–brain barrier [15], has intriguing pharmacological properties, inhibiting the function of α7 nicotinic acetylcholine (α7nACh) and N-methyl-D-aspartate (NMDA) receptors [16,17], serving as a ligand of GPR-35 [18], HCAR3 [19] and aryl hydrocarbon (AhR) [20] receptors, and activating M-type K^+^ channels [21]. By affecting one or more of these targets directly or indirectly, KYNA influences cholinergic and glutamatergic functions, which are critical for normal cognitive performance and are impaired in several major brain disorders [22,23,24,25]. Notably, the levels of KYNA have been consistently shown to be increased in the cerebrospinal fluid (CSF) and in the brain of people with schizophrenia (SZ) [26,27,28,29]. Elevated KYNA may therefore play a significant role in the pathophysiology of SZ, especially in the cognitive abnormalities observed in persons with schizophrenia [20].

Although TRP is rapidly degraded in the periphery, it also readily enters the brain via the large neutral amino acid transporter LAT-1 [15,30,31]. Within the brain, TRP normally serves as a bioprecursor of serotonin, which is further degraded to 5-hydroxyindoleacetic acid (5-HIAA) but is only poorly converted to kynurenine and its downstream metabolites [6,9,10]. In contrast, cerebral KP metabolism, including KYNA formation, is initiated, and largely controlled by blood-derived kynurenine. Notably, kynurenine, which, like TRP, uses LAT-1 to access the brain [32,33,34], can also function as an AhR agonist [35]. Importantly and in direct relation to elevations in the levels of proinflammatory cytokines, such as interferon gamma (IFN-γ), tumor necrosis factor alpha (TNF-α) and interleukin-6 (IL-6), KP metabolism is significantly affected by a compromised immune system [36]. Because of the presumed role of inflammatory processes in SZ and several other human brain diseases [37,38], this may affect the relationship between circulating and central TRP metabolites under pathological conditions [33,39,40,41].

In addition to urine analyses, which were commonly performed in the past and included examination of the effects of TRP administration in people with SZ [42,43,44,45], measurements in plasma have been increasingly used to investigate TRP metabolism in humans. These studies focused not only on the basal levels of TRP, 5-HT, 5-HIAA and various KP metabolites [46,47,48,49,50,51,52,53,54,55,56,57] but often also assessed the effects of acute TRP challenges in healthy individuals [58,59,60,61], as well as in people with SZ [48,59]. Although illness-related abnormalities were repeatedly reported in these studies, results have been inconsistent (see [62,63] for recent meta-analyses).

In view of the growing interest in the hypothesis that TRP metabolites, such as KYNA, play a significant role in the pathophysiology of SZ and of the still unresolved question as to whether the measurement of circulating metabolites can provide critical insights in this respect, we conducted a double-blind, placebo-controlled, crossover study to compare blood levels of three key metabolites of the two major branches of TRP degradation (kynurenine, KYNA and 5-HIAA) at rest and after an oral TRP challenge in healthy controls (HCs) and people with SZ to examine whether there are group differences in the conversion of TRP to serotonin or KP metabolites.

## 2. Results

Twenty-two participants with SZ and 16 HCs were recruited to and completed the study. No serious adverse events and no long-term changes in behavior or health were observed. Mean baseline plasma values were 1.6 ± 0.1 pmol/µL (kynurenine) and 26.0 ± 1.8 fmol/µL (KYNA) in HCs and 1.7 ± 0.1 pmol/µL (kynurenine) and 29.3 ± 3.3 fmol/µL (KYNA) in participants with SZ. The mixed model showed no significant difference in the baseline levels of either kynurenine or KYNA between the two groups (*t* = −1.37, *p* = 0.18 and *t* = −0.77, *p* = 0.45, respectively). The mean baseline values of 5-HIAA were 27.2 ± 1.6 fmol/µL in HCs and 35.3 ± 4.6 fmol/µL in participants with SZ, with no statistical difference between the groups (*t* = 1.4, *p* = 0.16). The basal serum levels of IFN-γ, TNF-α and IL−6 did not differ between HCs and participants with SZ (Table 1; *t* = −1.28, *p* = 0.21, *t* = 1.17, *p* = 0.25 and *t* = 0.48, *p* = 0.63, respectively).

**Figure 2:**
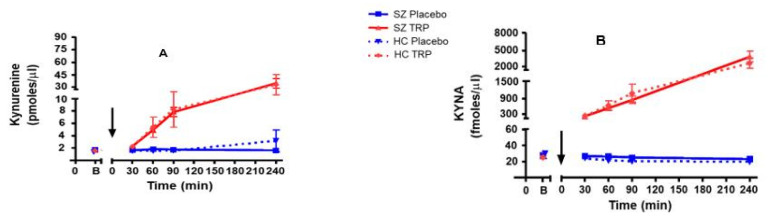

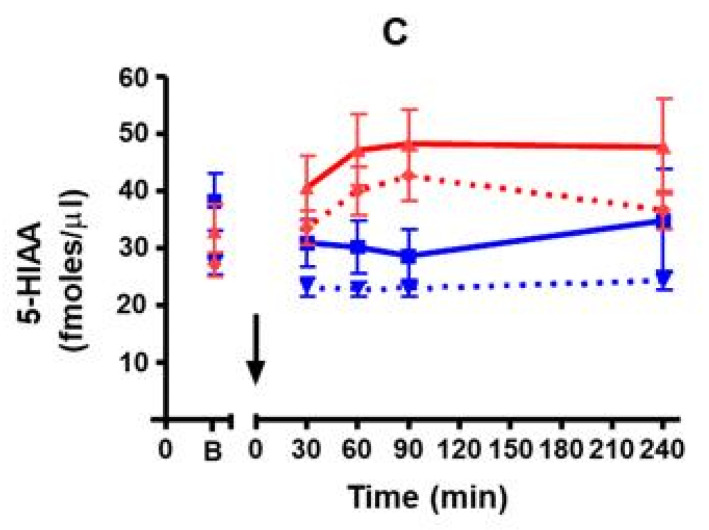
Levels of plasma kynurenine (**A**), KYNA (**B**) and 5-HIAA (**C**) at baseline and 30, 60, 90 and 240 min after oral administration of either placebo or TRP (6 g) in healthy controls (HCs, *n* = 16) and people with schizophrenia (SZ, *n* = 22) (arrows). Data are the mean ± SEM. Kynurenine (*p* < 0.001), KYNA (*p* < 0.001) and 5-HIAA (*p* = 0.038) increased after TRP challenge in both groups. See text for further details.

Assessment of samples obtained 30, 60, 90 and 240 min after the oral administration of TRP revealed rapid and time-dependent elevations in both kynurenine and KYNA, with the highest levels observed at 240 min (Figure 2A,B). The average increases in kynurenine and KYNA between basal values and the 240 min timepoint in HCs were 27-fold and 145-fold, respectively, and 21-fold and 135-fold, respectively, in participants with SZ. The mixed-model results showed that the effects of TRP on neither kynurenine (t = 12.20, *p* < 0.001) nor KYNA (t = 6.17, *p* < 0.001) differed between the two groups (t =−0.51, *p* = 0.61 and t = −0.80, *p* = 0.42). Placebo treatment did not affect the levels of the two KP metabolites in either group of study participants throughout the 4 h observation period. TRP raised 5-HIAA levels in both cohorts (t= −2.09, *p* = 0.038), causing a maximal increase of 1.4-fold in HC and 1.5-fold in participants with SZ after 240 min (Figure 2C), and the mixed model showed no difference in the effect of TRP between the two groups (t = 1.39, *p* = 0.165). Administration of placebo did not affect the levels of 5-HIAA at any timepoint in either group.

According to mixed-model examination, the TRP-induced increase in KYNA was larger than the increase in either kynurenine or 5-HIAA (t = 16.86, *p* < 0.001), and the kynurenine elevation was significantly greater than the increase in 5-HIAA (t = 3.58, *p* < 0.001).

To assess possible differences in the effectiveness of the two main branches of TRP degradation in HCs and participants with SZ, we compared plasma kynurenine/5-HIAA and KYNA/5-HIAA ratios between the two groups. These analyses revealed no statistical difference between the two groups in kynurenine/5-HIAA and KYNA/5-HIAA ratios after TRP administration (t = −1.23, *p* = 0.22 and t = 0.10, *p* = 0.92)**.**

Possible differential effects of TRP administration on the serum levels of IFN-γ, IL-6 and TNF-α in HCs and participants with SZ were examined using the linear mixed-effect model. No significant differences were observed as a result of the TRP treatment (Table 1; *t* = 0.05, *p* = 0.96; *t* = 0.82, *p* = 0.41; and *t* = 0.80, *p* = 0.43 for IFN-γ, TNF-α and IL-6, respectively).

## 3. Discussion

Using a double-blind, placebo-controlled, crossover study design, we determined the effect of TRP on the plasma levels of the KP metabolites kynurenine and KYNA, as well as the 5-HT metabolite 5-HIAA, in HCs and participants with SZ. These three compounds were selected to examine whether the peripheral dynamics of the two major pathways of mammalian TRP degradation differed between participants with SZ and HCs and whether measures of the peripheral dynamics may provide useful information regarding the proposed role of increased CNS KYNA levels in the pathophysiology of SZ. Measured both under baseline conditions and at four timepoints up to 4 h following a single oral challenge with TRP (notably prepared using cacao powder containing KYNA; [64]), the results showed, in agreement with the results of Badawy and Dougherty [58], the expected preferential and substantive conversion of TRP to the KP metabolites. However, our results did not reveal significant differences in kynurenine, KYNA or 5-HIAA levels or in the kynurenine/5-HIAA or KYNA/5-HIAA ratios between HCs and participants with SZ either before or after TRP administration.

A biological role of the essential amino acid TRP was first proposed more than a century ago [65], and it was soon recognized that several metabolic products of TRP serve not only physiological functions but may also be causally involved in the pathophysiology of various human diseases. Pioneering studies elaborated the biochemical events that account for and control the neosynthesis of a large number of biologically active TRP metabolites in mammals, with the kynurenine and serotonin pathways attracting the most attention [7,66]. Analyses of urine and blood samples from healthy humans consistently demonstrated that TRP, administered in large doses (usually 4–10 g) either orally or intravenously, is degraded by more than 90% via the KP, whereas only 5% or less is broken down to serotonin and beyond (see [44] for a review of early experiments in humans). Of relevance in the context of the present study, circulating TRP and kynurenine both readily enter the brain, whereas KYNA and 5-HIAA normally do not freely cross the blood–brain barrier [4,15,67]. Within the brain, TRP is an effective precursor of 5-HIAA but is only poorly converted to kynurenine [10]. Under physiological conditions, cerebral KP metabolism, including KYNA neosynthesis, is initiated primarily by kynurenine influx from the blood (cf. Introduction).

Although the present study is limited by the relatively small sample size and therefore does not provide sufficient statistical power to detect potential relationships to sex and age [58], our results—in line with recent meta-analyses [62,63]—demonstrate that steady-state plasma levels of kynurenine and KYNA cannot be reliably used as biomarkers of SZ or be assumed to reflect CNS kynurenine and KYNA levels (see below). This conclusion is likely unrelated to the effects of antipsychotic medication, as prolonged treatment with the antipsychotic risperidone affects neither the blood nor the brain concentration of KYNA in rats ([68] and K.V. Sathyasaikumar and R. Schwarcz, unpublished data), nor were there significant group differences in the baseline values of these KP metabolites. Notably, as previous reports have also dismissed effects of antipsychotics on circulating 5-HIAA [46,57,69], our observation that basal blood levels of 5-HIAA are in the normal range in persons with SZ suggests that this measure is of little value in terms of differentiating HCs from people with SZ. Further supporting the notion that immune activation is not consistently and specifically linked to KP dysfunction in SZ [39,62], we did not observe differences in the serum levels of IFN-γ, TNF-α or IL-6, i.e., three cytokines known to profoundly affect mammalian KP metabolism [36,70], between our two groups of study participants either at baseline or in response to the TRP challenge. These results are in line with a recent report that acute TRP *depletion* does not affect the levels of circulating cytokines, including TNF-α and IL-6, in humans [71].

Our results, showing expectedly large increases in plasma kynurenine and KYNA levels after the ingestion of 6 g of TRP in HCs and a much smaller conversion to the serotonin metabolite 5-HIAA, confirmed the results from similar previous bolus challenge studies [57,59,60,72,73,74]. In addition, the effects of orally administered TRP on all three metabolites did not differ significantly between HCs and persons with SZ. Together, these results show that neither basal nor stimulated levels of kynurenine, KYNA or 5-HIAA in the blood reflect or can be used to indicate pathological changes in TRP degradation in SZ. Although not the focus of the present study, the mechanisms underlying the maintenance of the substantive increases in circulating kynurenine and KYNA levels over the first few hours after an acute TRP challenge, as well as possible differential effects of these increases on peripheral organs in HCs and SZ patients, deserve attention and scrutiny in the future.

As the concentrations of both kynurenine and KYNA—but not of 5-HIAA [46,74,75]—have been consistently reported to be significantly elevated in the brain and CSF of people with SZ and given that these increases are believed to be involved in the pathophysiology of SZ [17,26,76,77,78], our results suggest that structural and/or functional abnormalities *within the brain*—rather than in the periphery—account for central KP impairments associated with schizophrenia. This may include changes in the blood–brain barrier [79], which could facilitate brain access of blood-derived kynurenine and, possibly, KYNA, as well as irregular astrocyte function [50,80]. The latter mechanism deserves special attention in light of the fact that astrocytes are the major source of newly synthesized KYNA in the brain [81] and because several *brain-specific mechanisms* control the formation and function of KYNA in these cells [82]. Methods to non-invasively monitor the fate and function of KYNA in the brain are still in development but should eventually allow investigators to explore these phenomena and use the new information for the benefit of people with SZ and beyond. Future studies should explore possible abnormal consequences of increased kynurenine levels with respect to AhR function [35] and of elevated levels of neuroactive kynurenine metabolites other than KYNA [12] in the brain of persons with SZ.

Although not likely to be valuable for clinical diagnosis of people with SZ, the reliable increase in metabolite levels in response to an oral TRP administration provides an excellent method for the examination and development of new drugs that target KP enzymes. Thus, in the context of the hypothesis that a reduction in brain KYNA levels provides clinical benefits to people with SZ [83], we are currently in the process of using this paradigm to evaluate the efficacy of brain-penetrant kynurenine aminotransferase II inhibitors, which have been repeatedly demonstrated to have remarkable pro-cognitive effects in relevant animal models [84,85,86,87], in both HCs and people with SZ. Together with parallel neuroimaging analyses and cognitive assessments, as well as the monitoring of possible group differences in functional effects on peripheral organs (see [88] for a recent comprehensive review), we expect this pharmacological approach not only to have promising therapeutic consequences but also to improve our understanding of the crosstalk of KP metabolites between the periphery and the CNS under physiological and pathological conditions in humans.

## 4. Materials and Methods

### 4.1. Study Participants

Participants were of either gender and of any race, with an age range of 18–65 years. Participants with SZ (*n* = 22) were recruited from the Maryland Psychiatric Research Center research clinics (Table 2). They met DSM-IV-TR (American Psychiatric Association: Diagnostic and Statistical Manual of Mental Disorders, Fourth Edition, Text Revision. Washington, DC: American Psychiatric Association, 2000) criteria for either SZ, schizoaffective or schizophreniform disorder. A best-estimate diagnostic approach was utilized, whereby information from the Structured Clinical Interview for DSM-IV [89] was supplemented with information from family informants, previous psychiatrists and medical records to generate a diagnosis. Participants with SZ were clinically stable, treated with the same antipsychotic for at least 60 days and were taking a stable dose of antipsychotic medication for the last 30 days. HC (*n* = 16) participants were recruited from the local community. They did not meet current SZ criteria and did not have a past history of a DSM-IV-TR Axis 1 diagnosis. Exclusion criteria included the following: Calgary Depression Scale total score ≥ 10 at baseline; current smoking (i.e., expired CO ≥ 10 ppm) or nicotine replacement therapy; pregnancy or breast feeding; and self-reported excessive daily caffeine use, defined as intake exceeding 1000 mg or the equivalent of 8 cups of coffee. Participants with active medical disorders known or suspected to affect TRP metabolism or to interfere with TRP absorption (e.g., acute intermittent porphyria, celiac disease, Crohn’s disease and irritable bowel syndrome) were excluded. Inclusion and exclusion criteria were evaluated at the initial screening visit.

The study (clinicaltrials.gov NCT02067975) was approved by the Ethics Committee of the Institutional Review Board (IRB) of the University of Maryland, Baltimore (UMB) (approval code: HCR-HP-00057861-8).

### 4.2. Study Design

Study participants were administered TRP and placebo in a randomized, double-blind, crossover design that was comprised of three visits. During the initial visit, all participants signed informed consent and completed screening and baseline assessments. This was followed by two challenge days, which were at least 2 weeks apart. Challenge day schedules included a morning fasting blood draw, TRP or placebo intake and four subsequent blood draws. In addition, all participants underwent clinical assessments for psychiatric symptoms, neuropsychological testing and post-challenge neuroimaging (data will be published elsewhere). Randomization was implemented with a permuted block design of 4, stratified by diagnosis (HC vs. SZ).

### 4.3. TRP Challenge

TRP is a dietary supplement, and its use is regulated under the FDA Dietary Supplement Health and Education Act (DSHEA) of 1994. TRP powder was obtained from Ajinomoto, North America, Inc. (Eddyville, IA, USA), and its use in the study was approved through the FDA (IND# 114931). TRP slurry was prepared by mixing 6 g of TRP with 7.8 g of Domino 10X confectioner’s sugar (Yonkers, NY, USA) and 6 g of Nestlé cocoa mix (Glendale, CA, USA). The dried mixture was placed into an 8 oz amber bottle. The placebo was prepared using lactose monohydrate powder and was matched to taste to the TRP slurry. At the time of dispensation, the mixtures were dissolved in 250 mL of distilled water, and the participant was instructed to drink the entire solution within a one-minute period. TRP and placebo mixtures were prepared and dispensed by a research pharmacist who did not participate in assessment procedures.

### 4.4. Blood Collection

Participants fasted overnight, and blood collection began at approximately 8 am. Protein-free snacks were provided soon after TRP or placebo administration. For the analysis of kynurenine, KYNA and 5-HIAA, blood samples were collected in EDTA-containing tubes 105 min before (baseline) and 30, 60, 90 and 240 min after the ingestion of TRP or placebo. After centrifugation (700× *g*, 10 min), the supernatant plasma was removed and stored at −80 °C until analysis. For the measurement of cytokines, blood was collected in a serum separator tube.

### 4.5. Kynurenine and KYNA Measurement

On the day of the assay, plasma samples were thawed and diluted 1:2 (*v*/*v*) in ultrapure water. A volume of 200 µL of the sample was deproteinized by the addition of 50 µL of 6% perchloric acid. After thorough mixing, samples were centrifuged (16,000 × g, 15 min), and 20 µL of the resulting supernatant was applied to a 3 µm ReproSil C18 HPLC column (100 mm × 4 mm; Dr. Maisch GmbH, Ammerbuch, Germany). Kynurenine and KYNA were isocratically eluted using a mobile phase containing 50 mM sodium acetate and 5% acetonitrile (pH adjusted to 6.2 with glacial acetic acid) at a flow rate of 0.5 mL/min. Using post-column derivatization with 500 mM zinc acetate delivered at a flow rate of 0.1 mL/min, the two KP metabolites were measured in the eluate via fluorimetric detection (kynurenine: excitation: 365 nm, emission: 480 nm; KYNA: excitation: 344 nm, emission: 398 nm; Series 200; Perkin-Elmer, Waltham, MA, USA). The retention times of kynurenine and KYNA were ~6 min and ~10 min, respectively.

### 4.6. 5-HIAA Measurement

A volume of 50 μL of plasma was mixed with 150 μL of ultrapure water and deproteinized by the addition of 50 μL 6% perchloric acid. Following centrifugation (16,000× *g*, 15 min) and recentrifugation of the resulting supernatant (again at 16,000× *g*, 5 min), the final supernatant was diluted (1:5, *v*/*v*) with ultrapure water, and 20 μL was injected onto a 3 μm HPLC column (BDS HyperSil; 150 mm x 3 mm; Thermo Fisher Scientific, Pittsburgh, PA, USA). 5-HIAA was eluted using a mobile phase containing 75 mM sodium monophosphate, 0.01% triethylamine, 25 μM EDTA, 1.7 mM octane sulfonic acid and 5% acetonitrile (pH 3.0) at a flow rate of 0.4 mL/min. In the eluate, 5-HIAA was measured electrochemically using an HTEC 500 detector (Eicom Corp., San Diego, CA, USA; oxidation potential: 450 mV) [90]. The retention time of 5-HIAA was ~12.5 min.

### 4.7. IFN-γ, TNF-α and IL-6 Measurement

For the determination of cytokines, 5 mL of blood was centrifuged within 30 min (10 min, 700× *g*), and the supernatant serum was immediately frozen at −80 °C. IFN-γ, TNF-α and IL-6 were analyzed by the Core Cytokine Laboratory of the University of Maryland (Baltimore, MD, USA).

### 4.8. Statistical Analysis

A linear mixed-effect model was used to examine possible differences between circulating levels of TRP metabolites and cytokines in HCs and people with SZ at baseline and after TRP administration. We also determined the kynurenine/5-HIAA and KYNA/5-HIAA ratios in all study participants (the data were log-transformed to approximate normal distribution) in order to assess differences in kynurenine and serotonin pathway activity between the two groups.

## Figures and Tables

**Table 1:** Circulating Cytokines.

**IFN-γ (pg/mL)**				
	HC Placebo	HC TRP	SZ Placebo	SZ TRP
Baseline	15.2 ± 4.6	14.8 ± 3.2	11.5 ± 0.5	11.3 ± 0.5
30 min	11.3 ± 0.8	11.4 ± 0.8	11.3 ± 0.4	10.6 ± 0.4
60 min	11.9 ± 1.1	11.2 ± 0.7	11.7 ± 0.6	11.3 ± 0.4
90 min	16.9 ± 5.4	11.8 ± 0.8	11.3 ± 0.5	11.4 ± 0.4
240 min	11.2 ± 1.0	10.7 ± 0.7	11.1 ± 0.4	10.9 ± 0.3
				
		**TNF-α (pg/mL)**		
	HC Placebo	HC TRP	SZ Placebo	SZ TRP
Baseline	12.4 ± 2.2	13.9 ± 2.2	12.9 ± 1.3	16.6 ± 4.3
30 min	11.4 ± 1.1	11.7 ± 1.3	14.0 ± 2.0	13.3 ± 1.2
60 min	11.4 ± 1.0	11.3 ± 0.9	13.1 ± 1.3	13.1 ± 1.4
90 min	14.0 ± 2.4	11.7 ± 1.1	12.9 ± 1.0	14.4 ± 1.8
240 min	11.3 ± 1.2	11.3 ± 1.0	12.1 ± 0.8	16.0 ± 3.1
				
		**IL-6 (pg/mL)**		
	HC Placebo	HC TRP	SZ Placebo	SZ TRP
Baseline	2.1 ± 0.2	2.4 ± 0.4	4.0 ± 1.2	3.1 ± 0.8
30 min	2.9 ± 0.8	3.1 ± 0.6	4.2 ± 1.2	3.0 ± 0.9
60 min	3.0 ± 0.8	3.0 ± 0.6	4.3 ± 1.2	3.0 ± 0.7
90 min	3.9 ± 1.2	4.8 ± 2.1	4.5 ± 1.2	3.7 ± 1.0
240 min	4.1 ± 1.8	3.1 ± 0.8	5.3 ± 2.0	5.2 ± 2.0

Serum levels of IFN-γ, TNF-α and IL-6 at baseline and 30, 60, 90 and 240 min after oral administration of either placebo or TRP (6 g) in healthy controls (HCs, *n* = 16) and people with schizophrenia (SZ, *n* = 21–22). Data are the mean ± SEM. There were no significant effects at baseline or after the TRP treatment (see text for details).

**Table 2:** Demographics of Study Participants.

	HC (*n* = 16)	SZ (*n* = 22)
**Mean age** (**years**)	34.9 ± 9.6	34.0 ± 11.1
**Median age** (**min, max**)	31.0 (21.7, 49.1)	29.9 (19.6, 53.3)
**Male/female** (***n***)	9/7 (56%, 44%)	13/9 (59%,41%)
		
**Race** (***n***)		
White	2 (12.5%)	7 (31.8%)
Black	12 (75%)	14 (63.6%)
Asian	1 (6.25%)	0
Mixed	1 (6.25%)	1 (4.6%)
		
**Medications** (***n***)		
Clozapine		8
Olanzapine		2
Paliperidone/Risperidone		5
Aripiprazole		4
Quetiapine		2
Thiothixene		1
		
Antidepressant		8
Anticholinergic		7

HC: healthy controls; SZ: participants with schizophrenia. Age is presented as the mean ± SD.

## Data Availability

Data is contained within the article.

## Abbreviations

CSF: cerebrospinal fluid; HC: healthy control; 5-HIAA: 5-hydroxyindoleacetic acid; 5-HT: 5-hydroxytryptophan (serotonin); IFN-γ: interferon gamma; IL-6: interleukin-6; KP: kynurenine pathway; KYNA: kynurenic acid; NMDA: N-methyl-D-aspartate; SZ: schizophrenia; TNF-α: tumor necrosis factor alpha; TRP: L-tryptophan.
